# Supplementary material for: Willingness and ability to pay for breast cancer treatment among patients from Addis Ababa, Ethiopia: A cross-sectional study
Source: PLoS One. 2024 Mar 28;19(3):e0300631. doi: 10.1371/journal.pone.0300631 (PMC10977721; doi:10.1371/journal.pone.0300631)
Supplement: Attachment — Submitted filename: Reviewer MA comments.docx [file pone.0300631.s001.docx]

**Comments:**

Overall, the abstract is well-structured with distinct sections for abstract, introduction, methods, results, and conclusions. This makes it easy to follow.

1. Line 59; consider rephrasing "According to the Addis Ababa Population Based Cancer Registry database" to "According to data from the Addis Ababa Population Based Cancer Registry."
2. Line 63, instead of "Many BC patients come to Tikur Anbessa Specialized Hospital-Radiation Centre," you could write "Many breast cancer (BC) patients seek treatment at the Tikur Anbessa Specialized Hospital-Radiation Centre."
3. Line 74; consider rephrasing "This holds true for BC patients" to "This is also applicable to BC patients."
4. Line 89, it would be clearer to replace "To determine whether the treatment is affordable or not" with "To determine the affordability of treatment."
5. Line 90, "Willingness to pay (WTP) is defined as a person's willingness to pay for the good or services he/she wants to buy while ability to pay (ATP) is defined as the person's ability to pay for the chosen good or services" could be simplified to "Willingness to pay (WTP) refers to a person's willingness to pay for desired goods or services, while ability to pay (ATP) refers to their financial capacity to do so.
6. Line 102; consider rephrasing "Examining WATP is central to a meaningful analysis of demand for health care services" to "Analyzing WATP is crucial for understanding the demand for health care services."
7. While you've mentioned that the sample size was distributed among the four health facilities based on the number of BC patients they treated before data collection, it would be beneficial to provide more details on how this distribution was carried out. Was it a simple proportional allocation, or were there other considerations involved?
8. It's good that you accounted for a non-response rate of 15%. However, providing a brief justification or citing literature for choosing this specific rate would enhance the transparency of your methodology.
9. The assumed standard deviation of direct medical costs ($355 USD per BC treatment) seems crucial for sample size calculation. Providing a justification or reference for this assumption would strengthen the validity of your calculations.
10. It's mentioned that imputations were not performed for missing data. While this is acceptable, it would be useful to mention the extent of missing data and any sensitivity analyses conducted to assess the impact of missing data on the results.
11. Line 114 "For this study, we included all BC patients who were permanently residing in Addis Ababa and who were receiving BC treatment in PCC and the three privately owned health facilities at the time of data collection." - To improve clarity, consider rephrasing: "For this study, we included all breast cancer (BC) patients permanently residing in Addis Ababa and receiving treatment at PCC and the three privately owned health facilities at the time of data collection."
12. Line 149 "Informed patient consent was obtained orally at the beginning of each interview and documented on the questionnaire by the data collectors." - Consider revising for clarity: "Informed consent was obtained orally from each patient at the beginning of the interview and documented on the questionnaire by the data collectors."
13. In Table 1 Explanation of Income Categories: Provide a brief explanation of the income categories (e.g., low-income, middle-income, high-income) to give context to readers who may not be familiar with the currency or income distribution in the study setting
14. In Table 4, the sample sizes for some categories (e.g., unemployed) are very small, which could affect the reliability of estimates.
15. It might be beneficial to compare the costs reported in this study with national or regional averages for breast cancer treatment. This comparison could help contextualize whether the observed costs are consistent with broader trends in healthcare pricing.
16. Line 271; there is number 3 between ( ); it stands for what?
17. Line 245; the numbers order is not corrected, Please fix it?
